# Seizure Action Plans and Health Care Utilization

**DOI:** 10.15844/pedneurbriefs-29-8-1

**Published:** 2015-08

**Authors:** Dara V.F. Albert, Anup D. Patel

**Affiliations:** 1 Division of Neurology, Department of Pediatrics, Nationwide Children's Hospital and The Ohio State University College of Medicine, Columbus, OH

**Keywords:** Epilepsy, Hospitalizations, Quality Improvement

## Abstract

Investigators from University of Utah School of Medicine studied the impact of a seizure action plan (SAP) on pediatric patients with epilepsy by measuring health care utilization as an outcome measure.

Investigators from University of Utah School of Medicine studied the impact of a seizure action plan (SAP) on pediatric patients with epilepsy by measuring health care utilization as an outcome measure. The study included 120 unique patients, 60 of which were utilized as historical controls. Patients were identified from the inpatient service and given a SAP prior to hospital discharge. The SAP used was an internally generated document containing key information such as the child's daily medications, emergency medications and Neurology provider contact. The patients were then followed for 18 months. Emergency Room (ER) visits, hospitalizations, clinic visits and Neurology office phone calls were tracked. They found no significant difference in ER or inpatient utilization between the patients who received an action plan and those who did not. This lack of difference persisted when they compared a subgroup of patients with less severe epilepsy, defined as less than 3 anticonvulsant medications. There was however, an increase in outpatient clinic visits in the group who received the SAP. The authors pose several possibilities as to why their results were not significant, such as a small cohort leading to insufficient power, the retrospective design and the format and layout of the SAP itself. They also suggest perhaps utilization was not the correct outcome measure. [1]

COMMENTARY. Pediatric epilepsy patients are complex and their families require an exceptional amount of education and support. Education has been shown to improve self-management and quality of life in adult epilepsy patients [2]. In pediatric patients, the child's self-perceived quality of life is strongly influenced by their social support system and less so their seizures [3]. In a recent meta-analysis, Ferro examined the literature for risk factors for health-related quality of life in pediatric epilepsy patients. His results also show that parental anxiety negatively impacted the child's quality of life [4]. Therefore, a quality of life metric may be more appropriate. In addition, the current study may be underpowered to detect changes in utilization based on the numbers of patients enrolled. Also, the patients enrolled in the study were selected from the inpatient service, which may have created a selection bias and limits the patient population. The outpatient arena may be more applicable for such an education driven intervention. The SAP developed is a text laden document. As the authors suggested, a color coded or less dense action plan might be easier to read and reference as what has been similarly used in other disease states, such as asthma. Despite the lack of significant findings in the current study, we believe educational tools such as a seizure action plan for patients with epilepsy and their families can still beneficial, the key will be determining the correct tool.
